# Acid-sensing ion channel 1a is involved in retinal ganglion cell death induced by hypoxia

**Published:** 2011-12-16

**Authors:** Jian Tan, Xinhai Ye, Yipin Xu, Hao Wang, Minjie Sheng, Fang Wang

**Affiliations:** 1 Department of plastic surgery, Shanghai Tenth People's Hospital, Tongji Unniversity, Shanghai, China; 2 Department of Ophthalmology, Shanghai Tenth People's Hospital, Tongji Unniversity, Shanghai, China

## Abstract

**Purpose:**

Loss of retinal ganglion cells (RGCs) during retinal ischemia is the potentially blinding mechanism that underlies several sight-threatening disorders. Fluctuations in extracellular pH are associated with such pathological conditions. It has been demonstrated that the retina is a functionally distinct region of central neurons that are known to contain acid-sensing ion channels (ASICs), which are depolarizing conductance channels directly activated by protons. This study was conducted to determine whether ASIC1a channels in RGCs are essential for ischemia-induced cell death.

**Methods:**

Expression of ASIC1a channels was detected in primary cultures of rat RGCs and in retinal sections. The patch-clamp technique in the conventional whole-cell configuration was used to examine the currents evoked by acid in the cultured RGCs. Intracellular Ca^2+^ ([Ca^2+^]i) elevation was detected by Ca^2+^ imagin*g*. Furthermore, hypoxia-induced cell death in RGC cultures was measured by methyl thiazolyl tetrazolium assay.

**Results:**

RGCs expressed a high density of ASIC1a channels. The expression and function of ASIC1a channels were upregulated after hypoxia in cultured RGCs. Ratiometric Ca^2+^ imaging showed that RGCs responding to a drop in pH presented an increase in the concentration of (Ca^2+^)i. Acute blockade of ASIC1a channels with the specific inhibitor amiloride or psalmotoxin 1 reduced RGC death in vitro.

**Conclusions:**

Based on these novel findings, we conclude that ASIC1a plays a role in RGC death induced by hypoxia. Therefore, neuroprotective strategies in glaucoma could include tools to improve the ability of these neurons to survive the cytotoxic consequences of ASIC1a activation.

## Introduction

Retinal ischemia is a serious and common clinical problem. It occurs as a result of acute vascular occlusion and leads to visual loss in several ocular diseases, including glaucoma, diabetic retinopathy, and hypertensive vascular disease. Transient global retinal ischemia shares many similarities with transient global cerebral ischemia [1-5]. Reperfusion after ischemia predisposes the retina to oxidative damage. Retinal ganglion cells (RGCs) have been reported to be particularly sensitive to acute, transient, and mild systemic hypoxic stress [6]. Loss of RGCs represents the final common pathway in the etiology of the disease [7]. In experimental studies, loss of RGCs and their axonal fibers has been demonstrated after retinal hypoxia [8,9].

Fluctuations in extracellular pH are associated with pathological conditions such as ischemia. The conduction of acid-evoked currents in central and sensory neurons is now primarily attributed to a family of proteins called acid-sensing ion channels (ASICs). ASICs are depolarizing conductance channels that are directly activated by protons. Four genes that encode seven subunits (ASIC1a, ASIC1b, ASIC1b2, ASIC2a, ASIC2b, ASIC3, and ASIC4) have been identified to date in mammals [10-15]. The ASICs belong to the degenerin epithelial Na^+^ channel superfamily. These channels are cation selective and sensitive to the diuretic amiloride [12]. They form homomultimeric and heteromultimeric cation channels. Moreover, the homomeric channel composed of ASIC1a is highly permeable to Ca^2+^ and Na^+^, whereas other homomeric or heteromeric ASICs are largely permeable to Na^+^ but impermeable to Ca^2+^ [13,16]. The homomeric ASIC1a channel is considered to be a nonclassical Ca^2+^ channel. ASICs are mainly expressed in the central and peripheral nervous systems, where they form homomultimeric and heteromultimeric cation channels.

There has been speculation about the physiologic and pathophysiological function of acid-gated currents in central neurons. It has been hypothesized that interstitial acidosis associated with seizures and ischemia could trigger their activity, thereby exacerbating the pathological consequences of these conditions [17]. The retina is a functionally distinct region of central neurons that contain epithelial Na^+^ channels [18,19], but it has only been demonstrated recently to contain ASICs [20,21]. Recent data have suggested that pH fluctuations play an important role in the retina. Although a few studies have indicated that ASIC1a is an important channel in normal retinal activity, no attempt has been made to establish its role in pathological changes. This has led us to examine the presence and functional role of ASIC1a in the retina during ischemia. We show that ASIC1a channels in RGCs are essential for ischemia-induced cell death.

## Methods

All experiments conformed to the ARVO Statement on the Use of Animals in Ophthalmic and Vision Research. For in vitro experiments, litters of Sprague-Dawley rats (Shanghai Institutes for Biologic Science, Shanghai China) were used at postnatal day (P) 3.

### Immunohistochemistry

Retinas were immediately fixed in 2% paraformaldehyde and cryopreserved, and frozen 10 µm thick sections were produced from similar eccentricities. Retinal sections were incubated overnight at 4 °C with sheep anti-ASIC1a antibody (1:100, a generous gift from Tianle Xu, Shanghai Jiao Tong University School of Medicine, Shanghai, China) or mouse anti-DNA-binding protein that identifies most mature neuronal populations (NeuN) monoclonal antibody, and developed with appropriate secondary antibodies conjugated with fluorescein-isothiocyanate.

### Immunopanning of retinal ganglion cells

RGCs from P3 rats were purified through sequential immunopanning to 99.0% purity, as previously described [22]. Briefly, the dissociated retinal cells from P3 Sprague-Dawley rats were incubated in flasks (Nunc A/S, Roskilde, Denmark) coated with an anti-rat macrophage monoclonal antibody (1:50) to exclude macrophages, and then incubated in tubes (Corning, Acton, MA) coated with an anti-rat Thy1.1 monoclonal antibody (1:300). RGCs adherent to the tubes were collected by centrifugation at 140× g for 5 min and seeded on 13-mm glass coverslips in a 24-well plate that had been coated with 10 μg/ml poly-l-lysine (Sigma-Aldrich, St. Louis, MO) and 1 μg/ml laminin (Invitrogen, Carlsbad, CA). Purified RGCs were plated at a density of approximately 1,000 cells/well. RGCs were cultured in neurobasal serum-free defined medium, which contained insulin (5 μg/ml), sodium pyruvate (1 mM), L-glutamine (1 mM), triiodothyronine (40 ng/ml; Sigma), N-acetyl cysteine (5 μg/ml; Sigma), and B27. Plates were incubated in a tissue culture incubator with humidified atmosphere containing 5% CO_2_ and 95% air at 37 °C for 12 - 14 days. RGCs were cultured on poly-D-lysine (70 kDa, 10 µg/ml; Sigma) and laminin (1 µg/ml; Invitrogen) in neurobasal serum-free defined medium, which contained insulin (5 µg/ml), sodium pyruvate (1 mM), L-glutamine (1 mM), triiodothyronine (40 ng/ml; Sigma), N-acetyl cysteine (5 µg/ml; Sigma), and B27.

### Electrophysiological recordings from isolated retinal ganglion cells

RGCs were maintained in low-density culture for 7–15 days. Whole-cell membrane currents were recorded via a single electrode using the patch-clamp technique. The standard external solution contained 150 mM NaCl, 5 mM KCl, 1 mM MgCl_2_, 2 mM CaCl_2_, and 10 mM glucose, buffered to various pH values with 10 mM HEPES (pH 6.0–7.4) or 10 mM 2-[N-Morpholino]ethanesulfonic acid (MES; pH <6.0), 300–330 mOsm/l. The typical resistance of the recording electrodes ranged from 4 to 9 M when filled with an intracellular solution that contained 120 mM KCl, 30 mM NaCl, 1 mM MgCl_2_, 0.5 mM CaCl_2_, 5 mM ethylene glycol tetraacetic acid (EGTA), 2 mM Mg-ATP, and 10 mM HEPES. The internal solution was adjusted to pH 7.2 with Tris base. Compensations of electrode capacitance and series resistance were used for all recordings. Recordings were performed at a holding potential of –70 mV using the Molecular Devices system (Axoclamp 200B, Digidata 1320A, pClamp 9; Molecular Devices, Foster City, CA).

### Ca^2+^ imaging

RGCs grown on 8×8 mm glass coverslips were washed three times with PBS and incubated with 1 µM fura-2-acetoxymethyl ester (Sigma) for 20 min at 37 °C; they were again washed three times and then incubated in standard extracellular solution for 30 min. Coverslips were transferred to a perfusion chamber on an inverted microscope (Nikon TE2000-E, Nikon, Melville, NY). Experiments were performed by using a 40× ultraviolet fluor oil-immersion objective lens, and images were recorded by a cooled CCD camera (Hamamatsu, Hamamatsu, Japan). The fluorescence excitation source was a 75 W xenon arc lamp. Ratio images were acquired by using alternating excitation wavelengths (340/380 nm) with a monochromator (Till Polychrome IV; TILL Photonics, Munich, Germany), and fura-2 fluorescence was detected at an emission wavelength of 510 nm. Digitized images were acquired and analyzed on a PC with SimplePCI (Compix, Inc., Brandywine, PA). Ratio images (340/380 nm) were analyzed by averaging pixel ratio values in circumscribed regions of cells in the field of view. The values were exported to Origin 7.0 for further analysis.

### Hypoxia-induced cell death in retinal ganglion cell cultures

Hypoxic stress was induced in vitro as described previously [23]. The cells were washed with Dulbecco’s modified Eagle medium without fetal bovine serum and then placed in the same medium in a hypoxic incubator (94% N_2_, 5% CO_2_, and 1% O_2_) for 2 h. Control cultures that were prepared using an identical passage of cells were simultaneously incubated in a regular tissue-culture incubator with 95% air/5% CO_2_ at 37 °C. At the end of the incubation period, cells were immediately subjected to experiments, which were performed in duplicate wells and repeated at least three times for each experimental condition.

### Lysate preparation and western blot

The cells were lysed in lysis buffer (25 mM Tris, pH 7.4,1% Triton X-100, 150 mM NaCl) with protease and phosphatase inhibitor cocktail (catalog no. 78428; Pierce, Rockford, IL). Protein concentration was determined by Bradford protein assay (Bio-Rad, Hercules, CA). Samples (30 µg) were separated by sodium dodecyl sulfate PAGE in 10% Tris-glycine precast gels and transferred to a polyvinylidene difluoride membrane (Millipore, Billerica, MA). The membrane was incubated for 1 h in blocking solution containing 3% BSA and 0.1% Tween-20, pH 7.6. This was followed by overnight incubation at 4 °C in the blocking buffer containing a sheep anti-ASIC1a antibody (1:500). Subsequently, the labeled proteins were visualized by incubation with a horseradish peroxidase–conjugated antisheep IgG (1:2,500; Santa Cruz Biotechnology, Santa Cruz, CA) followed by development with a chemiluminescence substrate for horseradish peroxidase (Pierce).

### Survival assays

Survival assays using methyl thiazolyl tetrazolium (MTT; Sigma) were performed as described previously [22]. RGCs were cultured in 96 well plates (2×10^3^ cells/well). After treatment, cells were supplemented with fresh medium containing 3-(5)-2, 5-diphenyl-tetrazolium bromide (0.456 mg/ml) and incubated for 1 h at 37 °C. The formazan found in viable cells was dissolved with 100 µl of dimethyl sulfoxide and determined by reading the OD in a microplate reader (Molecular Probes, Garching, Germany) at 550 nm. The optical density of formazan formed in control cells was taken as 100% viability. Absorbance was normalized with respect to the untreated control cultures to calculate changes in cell viability. MTT was dissolved in PBS at 5 mg/ml. This stock solution was added to the culture well (1:9) and incubated at 37 °C for 1 h. Viable cells with active mitochondria cleaved the tetrazolium ring into a visible dark-blue formazan reaction product. The density of each culture well was measured using a microplate reader (Molecular Probes, Garching, Germany) at 550 nm. The optical density of formazan formed in control cells was taken as 100% viability. Absorbance was normalized with respect to the untreated control cultures to calculate changes in cell viability.

### Statistical analysis

The control and the treated conditions were compared, and statistical significance at each time point was determined using one-way ANOVA. Data are presented as means±standard deviation (SD) from the three separate experiments. A p<0.05 was considered to be significant.

## Results

### Presence of acid-sensing ion channel 1a in retinal ganglion cells

**Figure 1:**
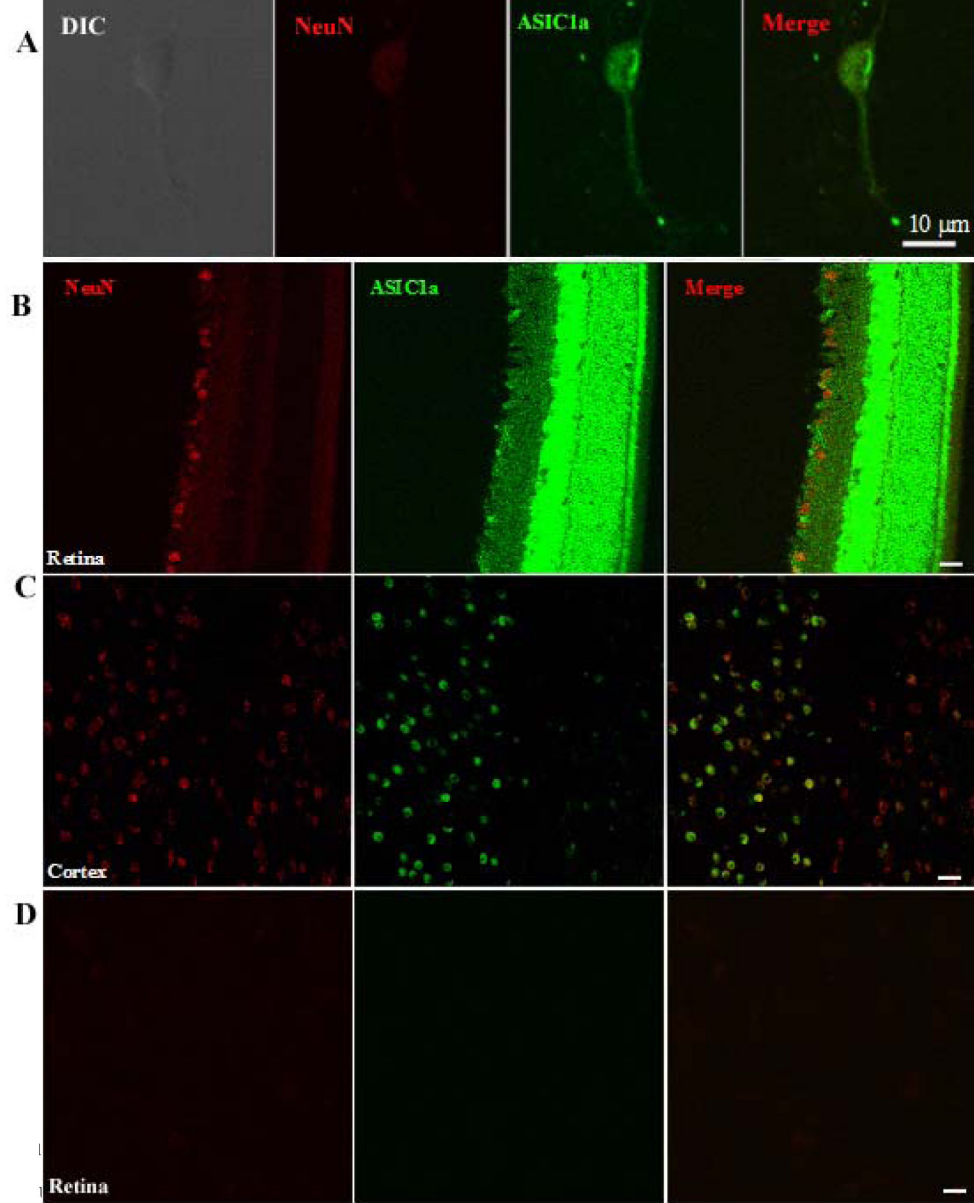
The presence of acid-sensing ion channel 1a in retinal ganglion cells in rat-cultured retinal ganglion cells and retinal slices using immunohistochemistry. **A**: Cultured retinal ganglion cells (RGCs) were identified routinely from other cell types on the basis of soma diameter (≥10 µm). Acid-sensing ion channel (ASIC) 1a immunoreactivity was colabeled with anti-DNA-binding protein that identifies most mature neuronal populations (NeuN) fluorescence. **B**: ASIC1a immunoreactivity was detected in rat retinal slices including the RGC layer, the inner segment of cone photoreceptors, the inner nuclear layer, the inner plexiform layer, and slightly in the outer plexiform layer. **C**: Positive control reactions of ASIC1a were performed using rat cortex slices. **D**: A negative control group for immunohistochemistry studies was conducted by removal of primary antibodies.

The presence of ASIC1a in RGCs was confirmed using immunohistochemistry in rat-cultured RGCs and rat retinal slices. Double labeling was done using an anti-ASIC1a antibody (Figure 1) and NeuN, a specific marker of RGCs. NeuN is a DNA-binding protein that identifies most mature neuronal populations, and recent studies have used NeuN as a marker for RGCs in the retina [24]. Cultured RGCs were identified routinely from other cell types on the basis of soma diameter (≥10µm) [25]. Meanwhile, the cells could be evaluated based on correlation with immunocytochemical staining of NeuN fluorescence. In cultured RGCs, ASIC1a immunoreactivity was colabeled with NeuN fluorescence (Figure 1A). Furthermore, Figure 1A shows that the immunoreactivity was mainly localized in the somata of RGCs and their neurites. Simultaneously, in rat retinal slices, ASIC1a immunoreactivity was detected in the RGC layer, the inner segment of cone photoreceptors, the inner nuclear layer, the inner plexiform layer, and slightly in the outer plexiform layer (Figure 1B). In the RGC layer, double staining with the NeuN antibody clearly showed the expression of ASIC1a in some RGCs. Positive control reactions were performed using rat cortex slices (Figure 1C).

### Acid-evoked currents in retinal ganglion cells

**Figure 2:**
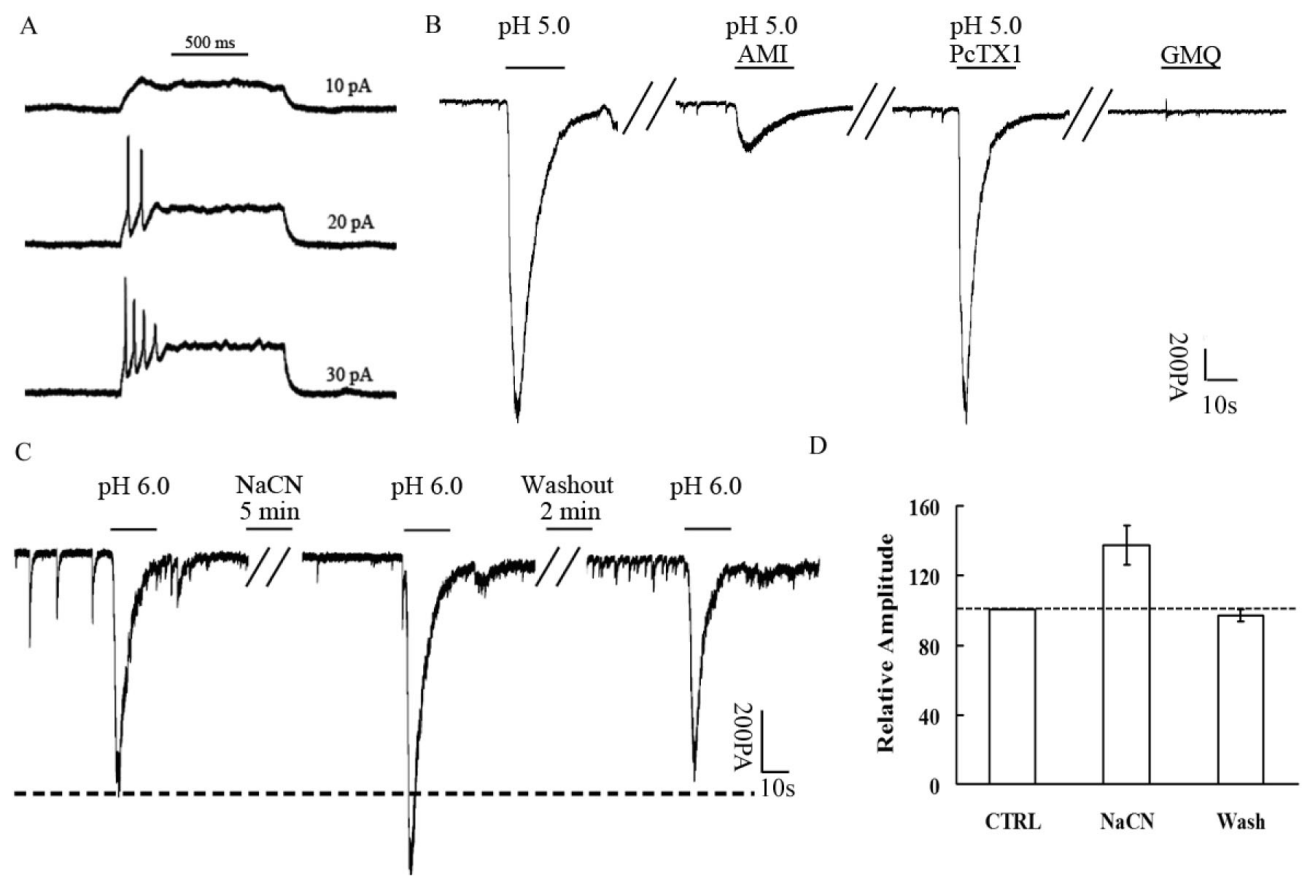
The currents evoked by acid in the rat cultured retinal ganglion cells using the patch-clamp technique. **A**: The retinal ganglion cells (RGCs) were able to trigger action potentials when stimulated. **B**: The typical current evoked by pH 5.0. This current could be blocked significantly by amiloride (100 µM) while it was not sensitive to 20 nM PcTx1, a specific antagonist of homomeric ASIC1a channels. Furthermore, the current was not evoked by GMQ, an agonist of homomeric ASIC3 channels. **C**: The amplitude of the current evoked by pH 6.0 changed during ischemia induced by NaCN treatment. The peak amplitude of current induced by pH 6.0 was potentiated after 5 min’ treatment with 100 µM NaCN (25±0.8% increase in the pH-6.0-induced current before NaCN treatment; n=5). **D**: Quantitative analysis of the peak amplitude of current induced by pH 6.0 before and after NaCN treatment. Data are mean±SEM (n=5 for each group). The asterisk indicates a p<0.05, compared with the ratio of control group.

To explore further the functional role of ASICs in RGCs, we examined the currents evoked by acid in the rat-cultured RGCs using the patch-clamp technique in the conventional whole-cell configuration (Figure 2). The cells were >10 μm in diameter and were able to trigger action potentials when stimulated (Figure 2A). RGCs can be identified from other cell types in the retina by these two characteristics. In these cells, we were able to evoke currents by pH 6.0 and pH 5.0 (Figure 2B,C). This current could be blocked significantly by amiloride (100 µM; Figure 2B), indicating that the current was mediated by ASIC activation. We also found that this native ASIC current was not sensitive to 20 nM (even to 200 nM) psalmotoxin 1 (PcTx1; Figure 2B), a specific homomeric ASIC1a inhibitor binding to its extracellular domain. Furthermore, we showed that the current was not evoked by 2-guanidine-4-methylquinazoline (GMQ), an agonist of homomeric ASIC3 channels. We investigated how the current changed during ischemia induced by NaCN treatment. We found that the peak amplitude of current induced by pH 6.0 was potentiated after 5 min treatment with 100 µM NaCN (25±0.8% increase in the pH-6.0-induced current before NaCN treatment; n=5; Figure 2C,D), a property reported to be characteristic of homomeric ASIC1a channels.

### Enhancement of the acid-induced (Ca^2+^)i elevation in cultured retinal ganglion cells

**Figure 3:**
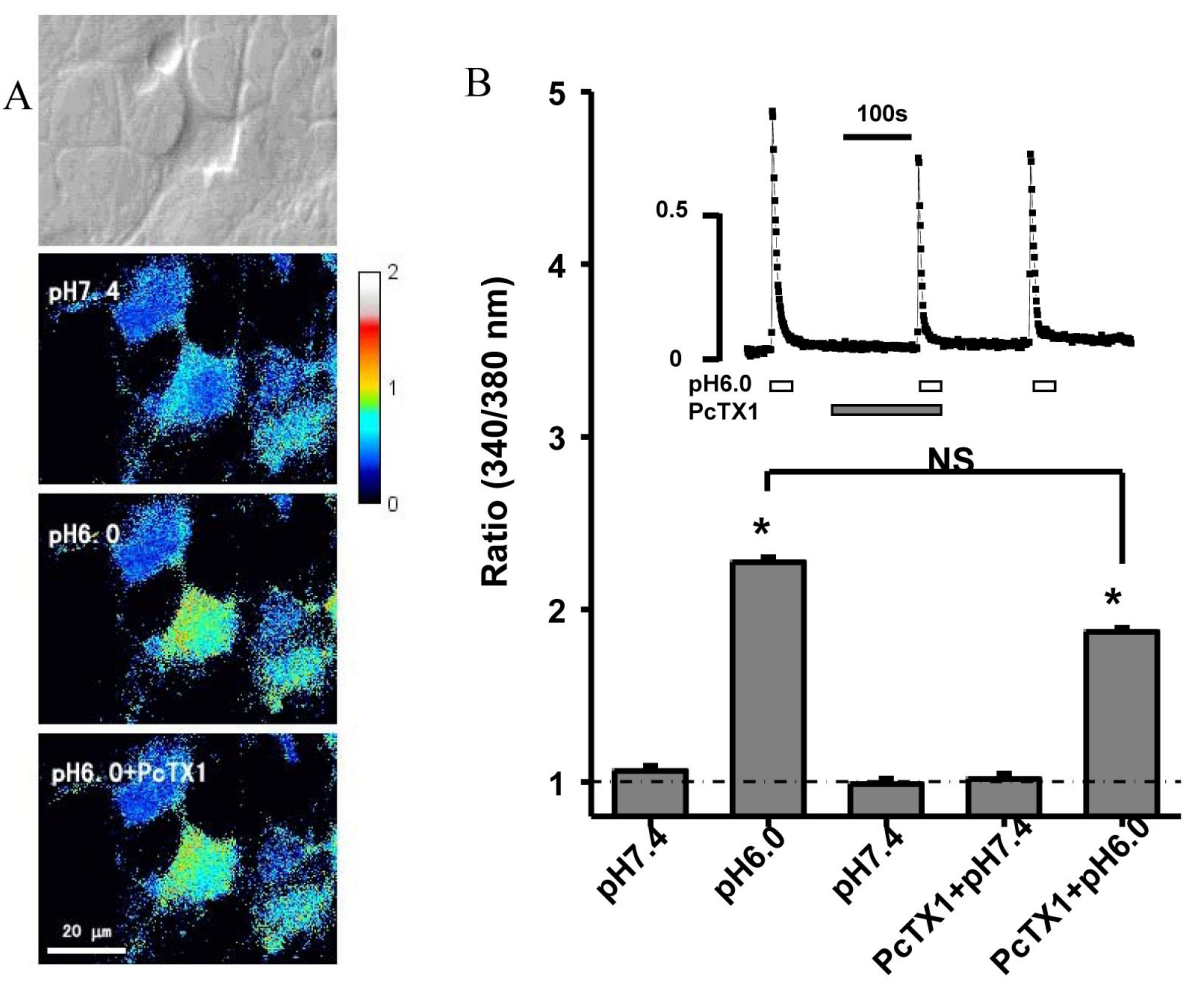
**A**: Representative images showing enhancement of the acid-induced [Ca^2+^]i elevation in cultured retinal ganglion cells (RGCs). **B**: Summary of results on the ASIC-mediated [Ca^2+^]i elevation. Dashed line indicates the basal level of fluorescence ratio (340/380). Inset, representative ratiometric measurements of [Ca^2+^]i elevation induced by a solution of pH 6.0. Data are mean±SEM (n=12 for each group). Asterisk indicates p<0.05, compared with the ratio induced by pH 7.4.

Consistent with the enhancement of ASIC current, ratiometric Ca^2+^ imaging showed that RGCs responded to a pH drop with an increase in the concentration of (Ca^2+^)i, an effect that was inhibited by 100 nM PcTx1 without statistical significance (Figure 3). Thus, we could only confirm that acid-induced enhancement of ASIC currents was accompanied by an increased acidosis-induced (Ca^2+^)i elevation.

### Retinal ganglion cell survival under hypoxic treatment

**Figure 4:**
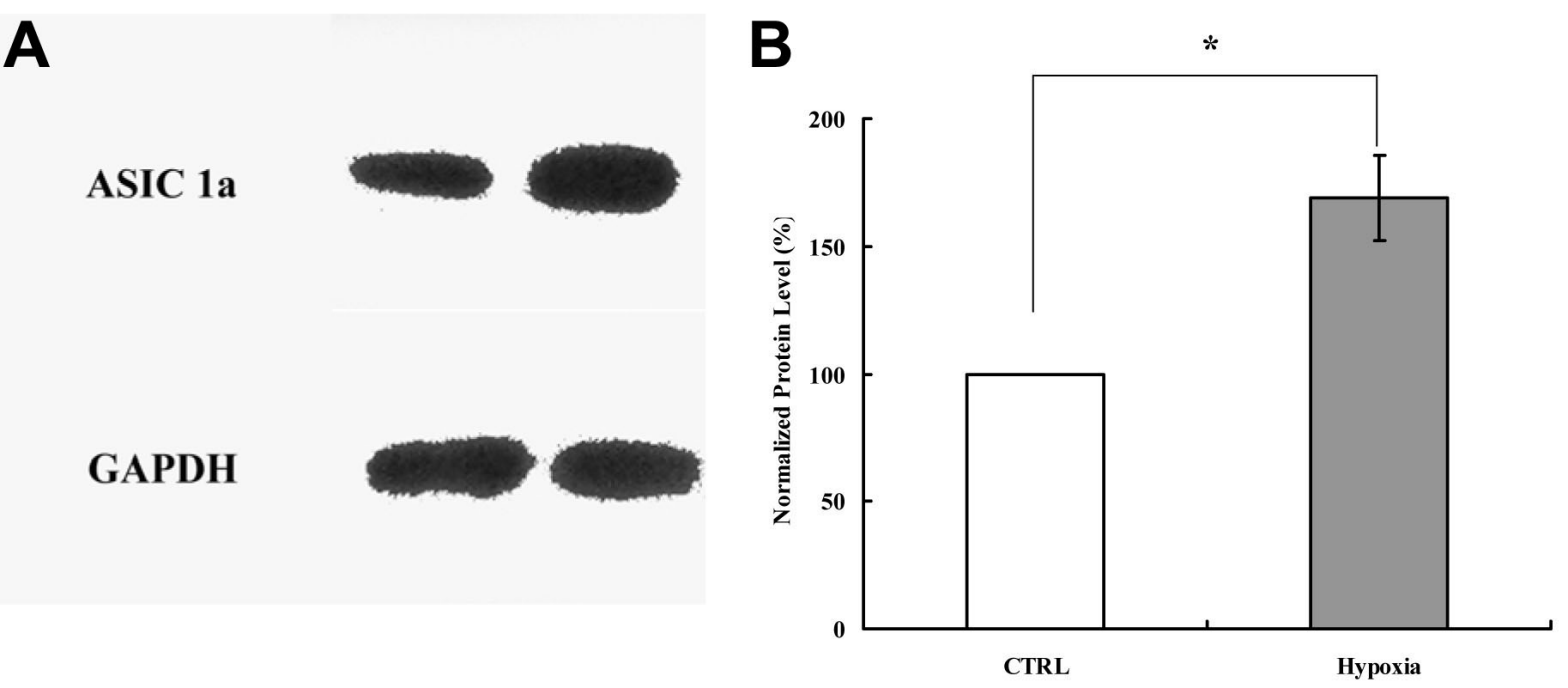
The expression changes of ASIC 1a in RGCs were detected using western blot following hypoxic treatment. Equal protein loading was assured by the use of a GAPDH-specific antibody on the same blot. **A**: Immunoreactive bands for ASIC 1a and GAPDH in control and hypoxic groups. **B**: Densitometry of the immunoreactive bands for ASIC 1a . Data are expressed as a percentage of the control value. Each column represents mean±SEM (n=3). Hypoxia significantly elevated the expression of ASIC 1a in the cultured RGCs. Asterisk indicates p<0.05, compared with the ratio of control group.

**Figure 5:**
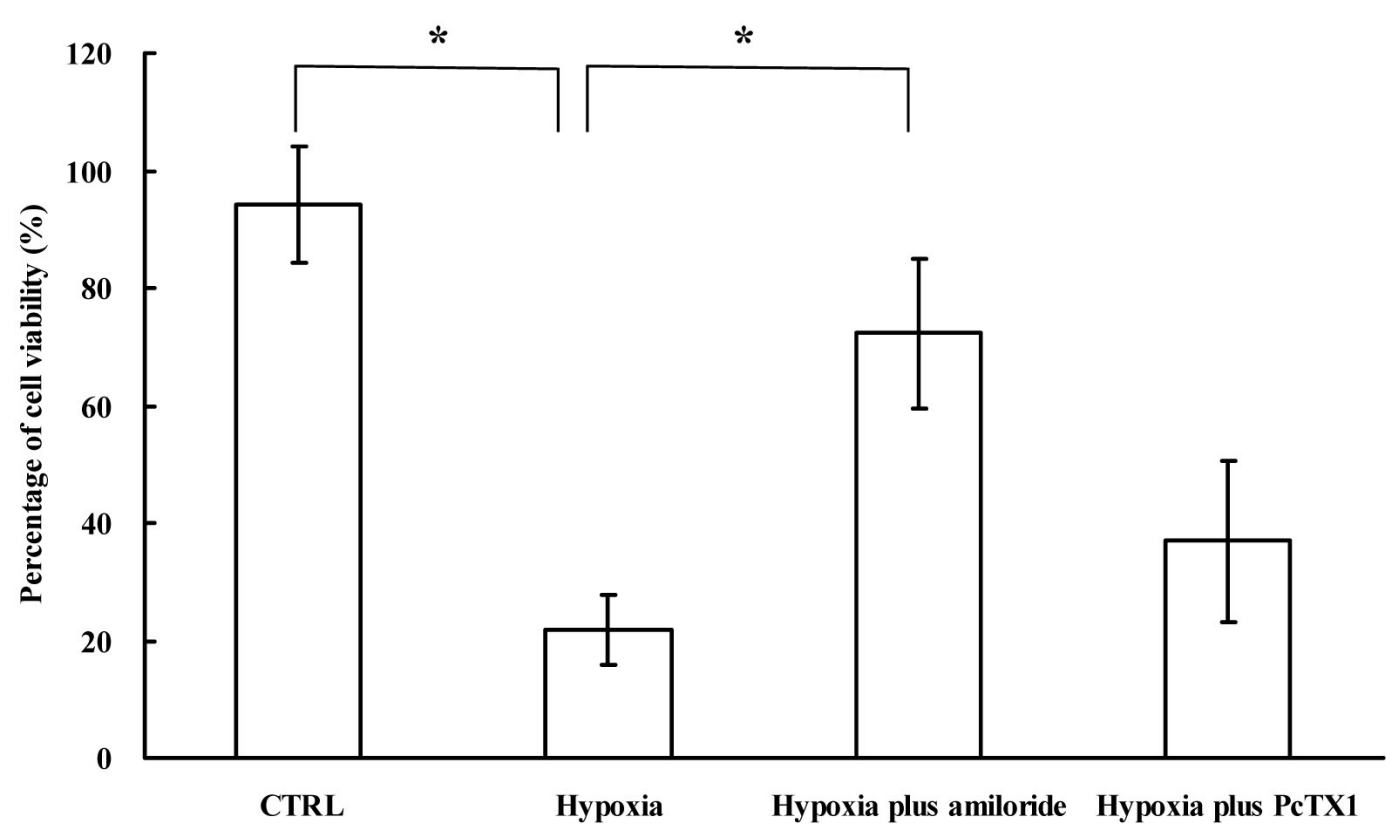
The effect of amiloride or psalmotoxin 1 on retinal ganglion cell death induced by experimental hypoxia in vitro. Hypoxia significantly reduced the survival of cultured retinal ganglion cells (RGCs) to 18.5±8.7% (n=3), whereas amiloride treatment increased RGC survival to 72.3±12.6% (n=3). Although psalmotoxin 1 (PcTX1) did not affect the current amplitude induced by acid under normal conditions, PcTX1 treatment increased RGC survival to 36.9±13.7% (n=3) after 2 h of hypoxia (*p<0.05).

The expression changes of ASIC1a in RGCs were detected using western blot following hypoxic treatment. The result indicated that the expression of ASIC1a was elevated significantly (Figure 4). The peak amplitude of the pH-6.0-induced currents in RGCs was potentiated after NaCN treatment, indicating that ASIC plays a role in retinal ischemia and RGC death. To test this possibility, we investigated the effect of amiloride or PcTX1 on RGC death induced by experimental ischemia and hypoxia in vitro. The RGCs were cultured from rats. Hypoxia significantly reduced the survival of cultured RGCs to 18.5±8.7% (n=3), whereas amiloride treatment increased RGC survival to 72.3±12.6% (n=3). Although PcTX1 did not affect the current amplitude induced by acid under normal conditions, PcTX1 treatment increased RGC survival to 36.9±13.7% (n=3) after 2 h of hypoxia (Figure 5).

## Discussion

In the present study, we demonstrated that ASIC1a channels in RGCs were essential for ischemia-induced cell death. This conclusion is based on the following evidence. First, RGCs expressed a high density of ASIC1a channels. Second, the expression and function of ASIC1a channels were upregulated after hypoxia in cultured RGCs. Third, acute blockade of ASIC1a channels with the specific inhibitor PcTX1 reduced RGC death in vitro.

It has previously been reported that ASIC1, 2, 3, and 4 mRNA was detected in the rabbit [26], mouse [25], and rat [27] retina. Few attempts have been made in previous studies to investigate the pathological role of ASICs in RGCs during retinal ischemia, although the subunit expression has been detected previously in the ganglion cell layer using immunocytochemistry, in situ hybridization [28-30], and electrophysiological recording. In this study, we confirmed that ASIC1a was expressed in rat-cultured RGCs and retinal slices using immunocytochemistry (Figure 1). In addition, we used electrophysiological recording to evaluate the functional role of ASICs in the RGCs. The currents evoked by rapid acidification were sensitive to amiloride (Figure 2), which indicated that ASICs were functionally expressed in the membrane of RGCs. We also found that the acid-evoked currents were not blocked by PcTX1 (a specific antagonist of homomeric ASIC1a channels), and GMQ (an agonist of homomeric ASIC3 channels) did not evoke an ASIC-like current in RGCs (Figure 2). The electrophysiological results in RGCs strongly suggest that the native ASIC current is not carried by a homomeric ASIC1a or ASIC3 channel. This conclusion is also supported in that PcTx1 does not inhibit the acid-induced (Ca^2+^)i increase by Ca^2+^ imaging (Figure 3). Combined with the relatively rapid kinetics of the native current (τ 471, 49 ms for the inactivation rate at pH 5, 50 mV), the native ASIC current might be supported by a heteromeric channel that is formed with ASIC1a, ASIC3, or a yet-to-be-described ASIC isoform, consistent with previous studies [31]. As far as the acid-induced (Ca^2+^)i increase in RGCs, we are investigating the source and mechanism of (Ca^2+^)i elevation in another work. There has been a discrepancy in previous studies about whether ASIC2a is expressed in RGCs, and this remains an open question. Remarkably, we found that the peak amplitude of ASICs evoked by acid increased after NaCN treatment (Figure 2), which is similar to the characteristics of ASIC currents in hippocampal [32] or cortical [17] neurons. Compared with the subunit expression in RGCs, ASIC1a and ASIC2a subunits have been shown to be abundant in the brain [33]. The homomeric ASIC1a channels contribute significantly to total acid-activated currents in cortical neurons, although coexistence of heteromeric ASIC1a/2a channels is possible. The results from previous studies suggest that the potentiation of ASIC activity induced by experimental ischemia is largely mediated by homomeric ASIC1a channels [34]. Although the subunit composition is different in RGCs in which there are mainly heteromeric ASIC1a channels, the ASIC currents still increase after experimental ischemia. Two possibilities may explain the discrepancy. First, ASIC1a forms homomeric ASIC1a channels on the membrane surface of RGCs through some unknown mechanisms; therefore, the ASIC currents are enhanced in RGCs in the same manner as in hippocampal or cortical neurons during ischemia. Second, the activity of heteromeric ASIC1a channels can be potentiated during experimental ischemia, similar to homomeric ASIC1a channels [35]. Homomeric ASIC1a channels are abundant in the cortical or hippocampal neurons and are permeable to Ca^2+^; therefore, they might cover the activity of heteromeric ASIC1a channels. However, we cannot exclude the possibility that the activity of heteromeric ASIC1a channels is modulated and plays a role in ischemia, especially when heteromeric ASIC1a channels dominate in RGCs.

To explore further the above possibilities, we tested the protective effects of PcTX1 against RGC death induced by experimental ischemia in cultured RGCs. We found that amiloride and PcTX1 had protective effects against RGC death induced by hypoxia. These results suggest that ASIC1a forms homomeric channels during ischemia, although the ASICs in RGCs were mainly heteromeric ASIC1a channels.

Our results suggest that ASIC1a is a novel mechanism that underlies ischemic RGC injury. Ischemic RGC injury plays a key role in a variety of retinal diseases such as diabetic retinopathy, hypertensive vascular disease, and glaucoma. Our findings could shed new light on the development of novel therapeutic neuroprotective strategies for these diseases.
